# Old Point Comfort

**Published:** 1897-07

**Authors:** 


### OLD POINT COMFORT.

The chairman of the Executive Committee of the American Dental Association furnishes the information that he has secured a rate of one fare and a third from the railroads, on certificate plan, and has also had promised from the proprietor of the Chamberlin Hotel a reduction from their regular charge to $2.50 per day, besides freely offering all the conveniences required for the meeting and committees. It is presumed, although not known by the writer, that the same will apply to the Hygeia, where the Association of Faculties is advertised to meet.

The Chamberlin has been recently constructed with all the modern appliances which add so much to the comfort of guests.

